# Comparison of Different Techniques for the Determination of Platinized Cytostatic Drugs in Urine Samples

**DOI:** 10.3390/molecules27238139

**Published:** 2022-11-23

**Authors:** Marina Arenas, Julia Martín, Juan Luis Santos, Irene Aparicio, Omar Fernández-Sanfrancisco, Esteban Alonso

**Affiliations:** 1 Departamento de Química Analítica, Escuela Politécnica Superior, Universidad de Sevilla, C/Virgen de África 7, E-41011 Sevilla, Spain; 2 Departamento de Medicina Preventiva y Salud Pública, Facultad de Farmacia, Universidad de Granada, E-18071 Granada, Spain; 3 Athisa Biogeneración, c/Periodista Francisco Javier Cobos, nº18, E-18014 Granada, Spain

**Keywords:** cytostatic drugs, cisplatin, carboplatin, oxaliplatin, LC-UV(DAD), LC-MS/MS, MRM mode, SIM mode

## Abstract

Platinum-based cytostatic drugs are one of the most widely used cancer treatments. They are excreted via the urinary tract and can reach the environment through wastewater, posing a risk to human health due to their side effects. Four identification and quantification techniques, including liquid chromatography (LC) separation coupled to (i) a diode array ultraviolet (UV(DAD)) (ii), mass spectrometer in single ion monitoring mode (LC-MS) and (iii) multiple reaction monitoring mode (LC-MS/MS) and (iv) derivatization with diethyldithiocarbamate prior to LC-MS/MS analysis, have been optimized and compared for the multiresidue determination of main platinized cytostatic drugs (cisplatin, carboplatin, and oxaliplatin) in urine samples. Parameters that affect the efficiency of the chromatographic separation and analytical determination of different methods (column, mobile phase, wavelength, precursor ions, fragmentor, and product ions) were optimized. Analytical features, such as matrix effect, sensitivity, precision, selectivity, and linearity, were calculated. In terms of selectivity, the derivatization technique was discarded since it was only applicable to the platinated sum. A high dilution of the sample with LC-UV(DAD) was needed to reduce the matrix effect. Overall, the LC-MS/MS method presented the best analytical features (% RSD ≤ 12.8%, R^2^ ≥ 0.991, or method-detection limits between 0.01–1 µg mL^−1^). The selected method was applied to the quantification of platinized cytostatic drugs in hospital urine samples from oncologic patients.

## 1. Introduction

Cancer ranks as a leading cause of death and an important barrier to increasing life expectancy in every country of the world [1]. Last year, there were an estimated nineteen million new cases around the world, and more than half of patients eventually die from it (GLOBOCAN 2020 database). Cytostatic drugs are frequently used to treat cancer and non-neoplastic illnesses [2]. Platinized antineoplastic drugs are among the most significant anticancer treatments and are successfully utilized to treat a variety of human cancers such as urologic, gynecologic, pediatric, testicular [3], lung, ovarian [4], and colon cancer [5]. They can be used alone or in combination with other cytostatic drugs. The most popular platinum-based medications are oxaliplatin (cyclohexane-1R,2R-diamine(oxalato)platinum(II)), carboplatin (*cis*-diammine(cyclobutane-1,1-dicarboxylate-O,O′)platinum(II)), and cisplatin (*cis*-diamminedichloroplatinum(II)) [3]. Their structure consists of a coordination complex containing a platinum atom in oxidation state II (Figure S1). These compounds interfere with the cell-division process by interacting with cell genetical materials. The mode of action consists in ligands being replaced by water molecules inside of the cells and producing reactive aquated complexes that could directly bind with DNA and inhibit cell divisions. However, this mechanism is non-selective and platinized drugs, as most cytostatic agents, may also affect non-tumor cells, which leads to genotoxic, mutagenic, and carcinogenic effects [6]. As a result, concern regarding their rate of use, their release into the environment, and their possible hazardous impacts on the ecosystem and human health has grown. Of particular concern are the risks to healthcare workers, who are exposed during drug preparation, the treatment, and disposal of medicines, as well as through contact with patient excrement. Recent works have studied the presence of cisplatin, among other cytostatics, as a potential health hazard in matrices present in hospital environments, such as air, surfaces, protective devices, and equipment [7,8,9].

Platinized drugs are eliminated relatively rapidly through the patient’s urine [6]. Cisplatin is excreted by this route by 28 ± 4% within 24 h of intravenous administration [10]; oxaliplatin urinary excretion is 53.8 ± 9.1% 5 days after administration [11] and carboplatin excretion rate is 93% during the first 3 days [12]. Due to the high excretion rate, patients’ urine is the main route by which platinized pollutants reach hospitals and municipal wastewaters [6], contaminating surface water, ground, and crop soils often irrigated with wastewater effluents or fertilized with sewage sludge or compost. 

As the main route of entry of these contaminants into the environment, it is of interest to develop analytical methods to determine the presence of these platinum-based pollutants both in biological and environmental samples. Different analytical methods have been proposed for the individual determination of these compounds [13] including phosphorescence in plasma and urine [14], atomic absorption spectrometry in plasma and mice organs [15], liquid chromatography (LC) coupled to an ultraviolet detector (LC-UV) in blood [16] and infusion solutions [17] or inductively coupled plasma mass spectrometry (ICP-MS) in plasma [18] and hospital wastewater [19]. However, these techniques have shortcomings regarding the multiresidue determination, since they have been applied to the determination of a single cytostatic (usually cisplatin) or total platinum, sensitivity, and selectivity. More recently, some LC methods using tandem mass spectrometry (MS/MS) determination have been developed to analyze carboplatin in plasma [20], carboplatin and oxaliplatin in surfaces [21] and cisplatin, carboplatin, and oxaliplatin in surfaces employing zwitterionic hydrophilic interaction liquid chromatography (HILIC) [22]. There are also studies that have proposed the determination of cisplatin or carboplatin indirectly through derivatization with diethyldithiocarbamate DDTC in plasma and urine [2,23,24,25]. Therefore, the aim of this study was to propose an analytical method for the simultaneous determination of cisplatin, carboplatin, and oxaliplatin in human urine. For that, four identification and quantification techniques, including liquid chromatography (LC) separation coupled to (i) a diode array ultraviolet detector (UV(DAD)) (ii), a mass spectrometer detector operating in single ion monitoring mode (LC-MS), and (iii) multiple reaction monitoring mode (LC-MS/MS) and (iv) derivatization with diethyldithiocarbamate previous LC-MS/MS analysis, have been compared and evaluated. 

## 2. Results

### 2.1. LC-UV(DAD) Method

To obtain the highest sensitivity and selectivity for the chromatographic separation in a shorter run time, the main variables affecting chromatographic separation and signal intensity were studied. First, a Zorbax Eclipse XDB–C18 Rapid Resolution HT (50 × 4.6 mm i.d.; 1.8 µm) and a HALO C-18 Rapid Resolution (50 × 4.6 mm i.d., 2.7 µm particle size) column were tested in order to obtain a high separation performance. The Zorbax Eclipse XDB–C18 Rapid Resolution column provided better peak shape in the shortest time with similar resolution for all the studied analytes. 

The effect of the mobile phase on chromatographic separation was also studied. Methanol (MeOH) and acetonitrile (AcN) were evaluated as organic modifiers (solvent B). As solvent A, deionized water with different additives was studied. The selection of the mobile phase was conditioned by the detector studied. With UV-DAD, a good chromatographic separation is key to identify the analytes besides the UV spectrum. The three platinates were separated in reversed phase using micellar liquid chromatography using 0.5 mM sodium dodecyl sulphate (SDS) as the aqueous phase and MeOH as the organic phase in isocratic mode (98:2, v/v). The flow rate was 0.6 mL min^−1^ and the total chromatographic run time was 6 min.

The compounds were identified by comparison of their retention times and UV spectra with those in the standard solution chromatograms. Peak areas were used for quantification. Calibration curves were built by linear regression of the peak areas of the standard solutions against their concentrations. 

**Figure 1:**
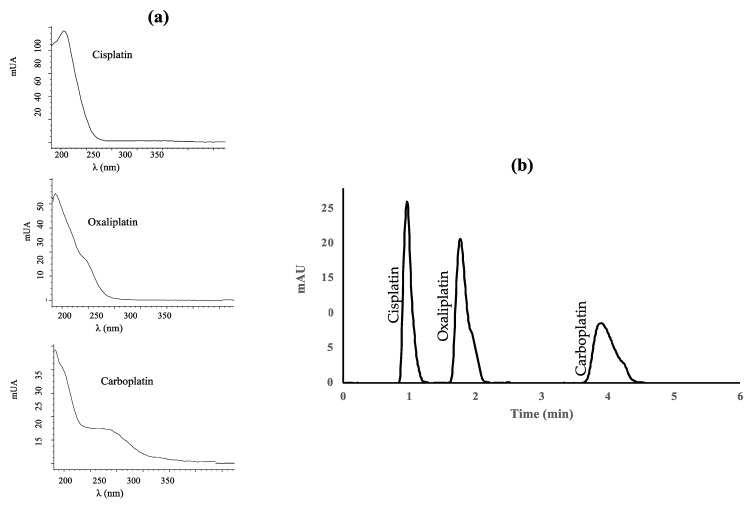
(**a**) Absorption spectra of cisplatin, oxaliplatin, and carboplatin; (**b**) Diode-array UV absorption chromatogram of a standard mixture containing 1 μg mL^−1^ of each platinized drug.

Platinum drugs were measured at the maximum absorption wavelength, except carboplatin, for which a compromise had to be reached between the wavelength providing the highest signal and the selectivity of the peak. The UV detector was set at 210 nm for cisplatin and oxaliplatin, and 254 nm for carboplatin. Figure 1 shows the UV absorption spectra of the platinum drugs studied and the UV(DAD) absorption chromatogram of standard mixture of the three platinized drugs under the optimal conditions. The peaks are well separated (resolution 2.5 for cisplatin-oxaliplatin and 5.2 for oxaliplatin-carboplatin).

### 2.2. LC-MS Methods

#### 2.2.1. SIM Mode

**Figure 2:**
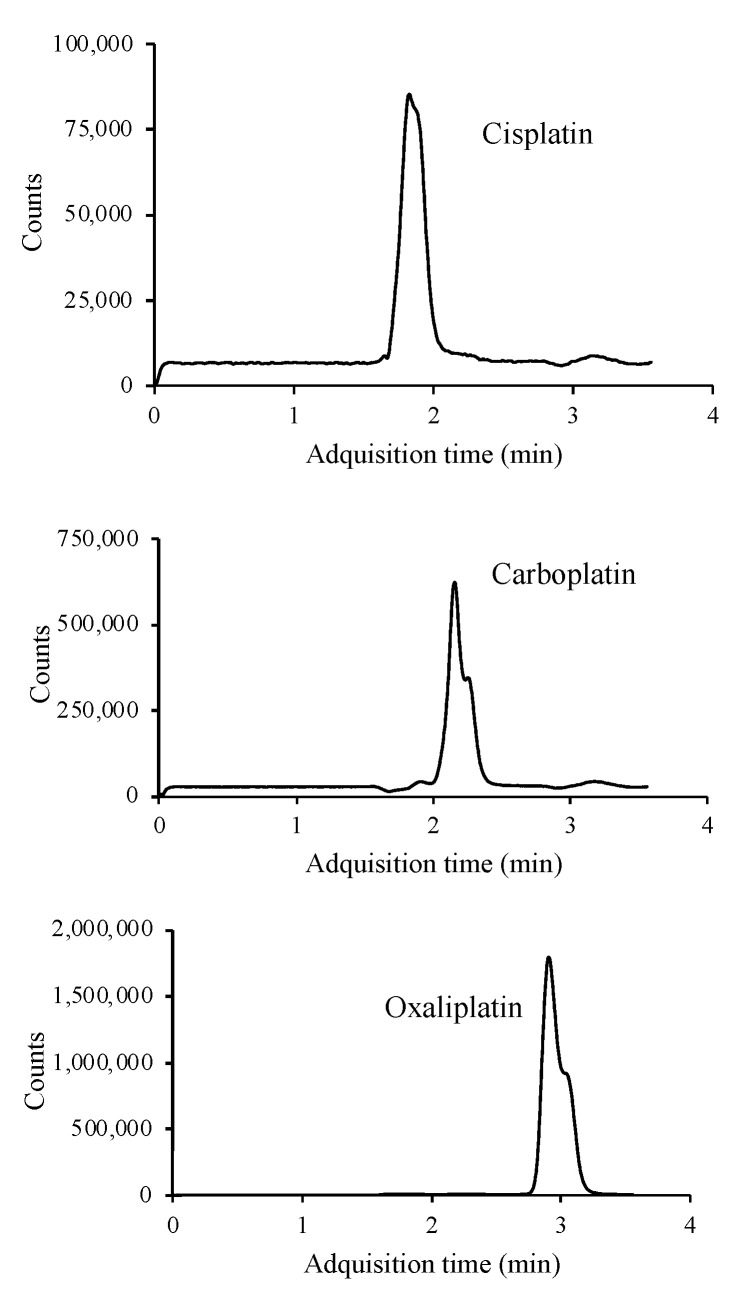
LC/MS chromatogram of a standard mixture containing 10 μg mL^−1^ of each of the platinum drugs studied.

The use of formic acid, acetic acid, ammonium formate, ammonium acetate and mixtures of these acids and the corresponding salts were assayed as additives in the mobile phase to improve the ionization of the target compounds and thus sensitivity. The platinized cytostatic drugs showed higher responses with better peak shapes using an isocratic elution with an aqueous buffer solution containing 20 mM ammonium formate (pH adjusted to 6.4 using formic acid) (95%) (A) and AcN (5%) as the mobile phase. The flow rate was 0.6 mL min^−1^ and the injection volume was 10 μL. Analysis was performed in 5 min. The MS detector was operated in SIM mode, where a precursor ion is sought to identify and determine the target compounds. The three platinized compounds were ionized in positive mode, with [M + NH_4_]^+^ being the most abundant molecular ion for cisplatin (318 m/z) and [M + H]^+^ for oxaliplatin (398 m/z) and carboplatin (372 m/z) (Figure S3). Overall, the abundance of the precursor ion of cisplatin is lower than for carboplatin and oxaliplatin precursor ions. Figure 2 shows the SIM chromatogram of a standard mixture of the three platinized drugs under the optimal conditions.

#### 2.2.2. MRM Mode

**Figure 3:**
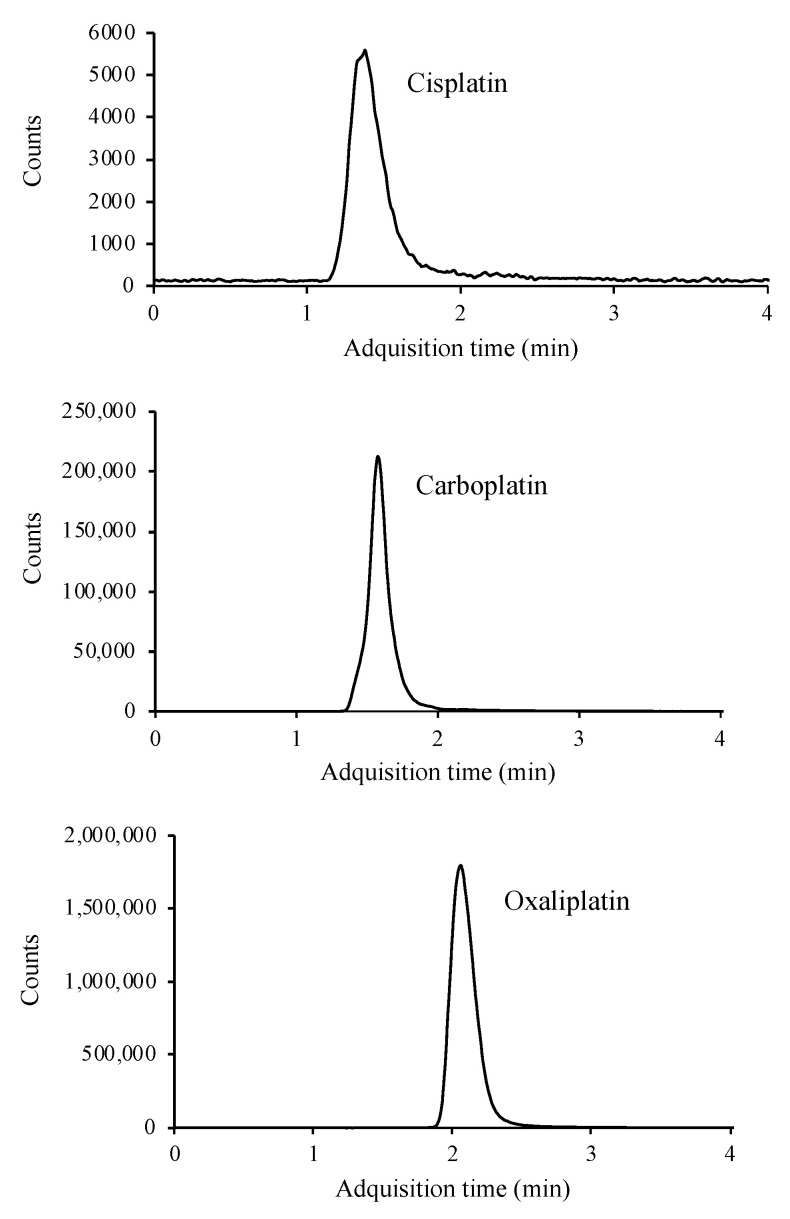
LC/MS-MS chromatogram of a standard containing 10 μg mL^−1^ of cisplatin, carboplatin, and oxaliplatin.

Chromatographic conditions were the same as described for SIM mode. The MS detector was operated in MRM mode, where product ions after fragmentation are used for quantitative purposes. Optimized LC-MS/MS parameters for the determination of platinated compounds are given in Table 1. Adequate fragmentation is achieved for carboplatin and oxaliplatin. However, cisplatin presents a low fragmentation in the collision cell. This issue, added to the matrix effect when applying the method to real samples, results in a small signal in the chromatogram. To reduce the matrix effect and improve the signal intensity, different dilutions were applied to the matrix, selecting 1:10 as the most optimal. Figure 3 shows the MRM chromatograms.

This technique, like the previous ones, allows separation in less than 4 min, as well as simultaneous determination of the three compounds. 

#### 2.2.3. Derivatization and MRM Mode

To improve sensitivity, derivatization with DDTC was proposed following a slightly modified procedure as previously described [23]. For sample derivatization, a 0.1 N NaOH solution was prepared containing 5% DDTC. Platin derivatives were prepared by adding 100 μL of a 5% DDTC solution to 500 μL of urine. Samples were homogenized by vortex for 15–20 s and incubated for 15 min at 45 °C [23]. In these incubation conditions, the sulfur of DDTC reacts with hydrated platinum to form the Pt-DDTC complex (Figure S2 Supplementary Material). After incubation, 1400 μL of acetonitrile are added to the sample to precipitate the protein. The sample was vortexed for about 15–20 s, and then placed into a microcentrifuge for 15 s at 14,500 rpm. An aliquot of the upper layer was injected into the LC-MS/MS, previous filtration through a 0.22 μm nylon filter.

Determination of derivatized compounds was carried out by LC-MS/MS. The mobile phase included A: acetonitrile (formic acid 0.1% v/v) and B: 10 mM ammonium formate (formic acid al 0.1% v/v). Separation was performed under the following gradient elution program: 0–0.5 min 5% A, 0.5–1 min 5–75 %A, 1–1.5 min 75–90% B, 1.5–2.5 min 90–95% A, 2.5–4 min 95% A, 4–5 min 95–100% A, 5–6 min 100% A, 6–7 min 100–5% A, 7–11 min 5% A. The injection volume was 10 µL and the column was set at a temperature of 35 °C. The flow rate was maintained at 0.4 mL min^−1^. The MS detector was operated in MRM mode. Optimized MS/MS parameters are shown in Table 2.

**Figure 4:**
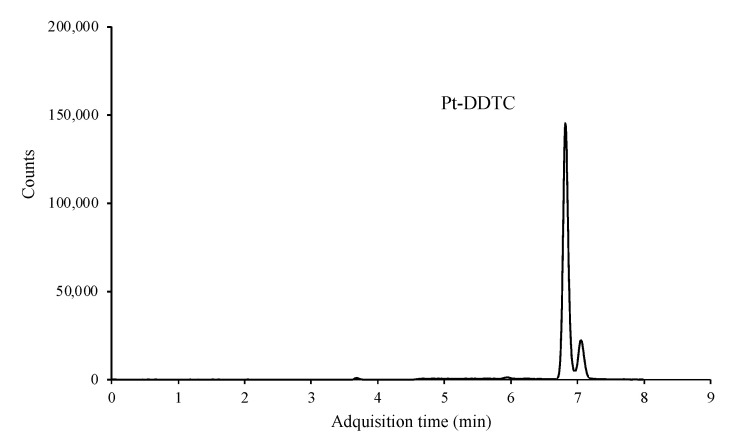
LC/MS-MS chromatogram of a standard mixture containing 0.1 μg mL^−1^ of each of the platinized compounds after derivatization.

Two product ions, one for quantification and other one for confirmation, were monitored. However, this method has no obvious benefit in terms of selectivity, since the three platinized compounds formed the same adduct, binding only and exclusively to the platinum atom. Therefore, and although the sensitivity of the technique was excellent, being able to reach a few μg mL^−1^, it was impossible to differentiate between the three platinized compounds and only the peak sum of the three adducts was observed (Figure 4).

## 3. Discussion

The chromatographic conditions used in each method were conditioned by the detector employed. With UV-DAD, a good chromatographic separation is key to identify the analytes besides the UV spectrum. The three platinized cytostatic drugs were perfectly separated in reversed phase using micellar liquid chromatography, with 0.5 mM SDS as the aqueous phase and MeOH as the organic phase in isocratic mode. In the MS/MS detector, however, compound identification was achieved, besides its retention time, using the ratio between two mass transitions (qualifier and quantifier). In most of cases, the criteria used are those proposed by Commission Decision 2002/657/EC [26] which involves matching ±2% of retention times and 80% agreement in relative ion ratios. Due to the higher selectivity of MS/MS detector, the separation of analytes is not so critical as occurs in UV-DAD. In this case, instead of a surfactant as mobile phase, the use of an isocratic elution with an aqueous buffer solution containing 20 mM ammonium formate (pH adjusted to 6.4 using formic acid) (95%) (A) and AcN (5%) as the mobile phase was selected to improve the ionization of the precursor ions of the target compounds and thus sensitivity.

On the other hand, the use of internal standards is a common practice used to compensate the signal enhancement or suppression known to occur in electrospray ionization (ESI). It could not be used in this study since the percentage of matrix effects observed was not the same for all the compounds. As such, a matrix-matched calibration curve method was used to overcome the matrix effect. In the case of the DAD method, because of the high volume of sample injected, the use of an internal standard was not necessary. Since all proposed techniques involve the direct injection of urine samples, the influence of the matrix effect was evaluated by comparison of calibration curve slopes in pure solvent (external calibration curves) and calibration curve slopes in urine (matrix-matched calibration curves). Calibration curves were prepared in triplicate at five concentration levels in a range from method quantification limits (MQLs) to 100 μg mL^−1^ and were constructed using analyte area (axis y) versus analyte concentration (axis x). Student’s t test was applied to evaluate statistical differences between external calibration and matrix-matched curves. The t calculated was compared with the two-tailed tabulated value, t_tab_ for the appropriate number of degrees of freedom at P% confidence. Typical values are k = 2 for 95% confidence [15], so, if t < k, the ratio of the slopes is not significantly different from 1, and, if t > k, the ratio of the slopes is significantly different from 1 and the matrix calibration must be used. The calculated Student’s t (2.93–14.8) showed statistical differences among the slope values of the calibration curves and, consequently, the use of matrix calibration was necessary. 

The analytical features of the proposed and optimized methods were calculated and compared (Table 3). Linearity was evaluated in terms of R^2^, preparing matrix calibration curves in triplicate in the range from the MQL to 100 μg mL^−1^. Precision was calculated as relative standard deviation (RSD%) for spiked samples in triplicate and selectivity was evaluated as the ability to produce results that depend only on the analyte to be identified or quantified, without interference from other species present in the sample. MDL and MQL were determined as the concentration, with a signal-to-noise ratio of 3 and 10, respectively.

In terms of linearity, correlation coefficients were higher than 0.992 for all the methods. Precision, expressed as RSD%, was less than 15% for all methods. 

Selectivity is adequate only for methods employing LC coupled to MS. With the UV-(DAD) detector, the drawback found was twofold: on the one hand, the platinized compounds do not present a characteristic absorption spectrum that would allow an unequivocal identification, and, on the other hand, a high loss of selectivity caused by the presence of interferences was observed when the method was applied to urine samples. Urine matrix had to be highly diluted (1:50) to avoid loss of selectivity and to be able to quantify the samples. In the method employing derivatization, the DDTC binds exclusively to a platinum atom, which means that the three analytes form the same precursor. Derivatized cisplatin, carboplatin, and oxaliplatin result in a single adduct, so determination of the individual analytes is not possible. 

The lowest MQLs are achieved with the derivatization method while the UV(DAD) method has the highest one due to the large dilution of the sample that was necessary to reduce the matrix effect. In the MRM method, for carboplatin and oxaliplatin drugs there is a great improvement in sensitivity compared to the SIM mode (from 7.5 to 0.50 and from 2.5 to 0.05 μg mL^−1^, respectively). Therefore, according to the analytical features of the proposed techniques, the best determination conditions are achieved by LC-MS/MS MRM mode. 

As the Introduction states, previously published works are focus on the single determination of a platinized compounds, mainly cisplatin, which may lead to lower quantification limits (especially if a derivatization step is being used, as can be seen in our results in Table 3). To the authors’ knowledge, there is only one published work that achieves this aim. Recently, Dugheri et al. (2021) developed a method for the determination of cisplatin, carboplatin, and oxaliplatin in surfaces. However, besides being applied to a completely different type of sample, it employs zwitterionic hydrophilic interaction liquid chromatography. This technique presents some advantages, such as a higher sensitivity than in reversed-phase LC when it is used with electrospray ionization mass spectrometry. Despite this, it also presents some shortcomings. For example, matrix effect in biological samples is expected to be more pronounced in HILIC than in reversed-phase LC due to the presence of phospholipids, which are eluted close to the peaks of interest in HILIC. Furthermore, the use of HILIC is not as extended as LC in reverse mode. 

## 4. Materials and Methods

### 4.1. Chemicals and Reagents 

Analytical quality standards (<99%) of carboplatin, cisplatin, and oxaliplatin were purchased from Sigma-Aldrich (Steinheim, Germany). LC-MS-grade acetonitrile, MeOH, and water were supplied by Romil (Barcelona, Spain). Formic acid (98%) was provided by Panreac (Barcelona, Spain). Analytical-grade SDS, sodium diethyltiocarbamate trihydrate, and ammonium formate were provided by Sigma-Aldrich (Madrid, Spain). NaOH was purchased from Panreac (Madrid, Spain). 

### 4.2. Standard Solutions

Stock standard solutions of carboplatin and oxaliplatin were prepared in MeOH:H_2_O at 1000 µg mL^−1^ and standard solution of cisplatin was prepared in water 0.9% KCl (p/v). Each standard solution was kept at −18 °C in amber glass bottles. Working solutions were made by diluting or mixing individual standard solutions to produce mixtures of target compound.

### 4.3. Instruments

#### 4.3.1. LC Coupled to UV-DAD Detector

The LC-UV(DAD) system consisted of a high-performance liquid chromatograph Agilent 1200 series (Agilent, Santa Clara, CA, USA) with high-pressure binary pump, autosampler, and column oven coupled to a UV-DAD detector. Chromatographic separation was performed on a Zorbax Eclipse XDB C-18 Rapid Resolution (4.6 × 50 mm i.d., 1.8 µm) column (Agilent, Santa Clara, CA, USA) thermostated at 30 °C.

#### 4.3.2. LC Coupled to a MS Detector

The LC-MS system was composed of a c with high-pressure binary pump, autosampler, and thermostated column compartment, coupled to an Agilent 6495 series HPLC (Agilent, Santa Clara, CA, USA) coupled to a 6410 triple quadrupole (QqQ) mass spectrometer with electrospray ionization. 

Chromatographic separation was carried out chromatographic separation using a HALO C18 (50 mm × 4.6 mm d.i., 2.7 μm) column thermostated at 30 °C and coupled to a HALO C18 (5 mm × 4.6 mm d.i., 2.7 μm) guard column.

MS detector was operated using electrospray ionization in the positive mode with the spray voltage set at 4000 V. Nitrogen was used as nebulizer gas, and nebulizer pressure was set at 40 psi with a source temperature of 250 °C. Drying gas (nitrogen) was heated to 350 °C and delivered at a flow rate of 12 L min^−1^.

### 4.4. Sample Collection and Treatment

Urine samples were collected from hospital and belonged to oncology patients treated with different cytostatic drugs. Samples were directly injected, after filtration through a 0.22 μm nylon filter, except for those that required a prior derivatization step.

## 5. Conclusions

Four determination techniques, including LC separation coupled to UV-(DAD), MS in SIM and MRM mode, and derivatization with DDTC followed by MS/MS, were optimized for the analysis of the main platinized cytostatics (cisplatin, carboplatin, and oxaliplatin) in urine samples. The LC-MS/MS method showed the best results (R^2^ < 0.991, RSD% < 13, good selectivity, and MDL < 1 µg mL^−1^). UV-(DAD) and derivatization methods have no obvious benefit in terms of matrix effect and selectivity, respectively, since in the first case the matrix effect prevented quantification in real samples and, in the second case, the three platinized compounds formed the same adduct. Despite the limitations of having poorer selectivity, the derivatization method offers the advantages of its high sensitivity being able to be used for an individual and specific analysis.

## 6. Application

The LC-MS/MS method was applied to real urine samples belonging to patients under treatment with platinized drugs. Results can be seen in Table 4. Cisplatin, carboplatin, and oxaliplatin were detected in the samples in concentrations ranging from 0.331 to 58.7 µg mL^−1^.

## Figures and Tables

**Table 1:** Optimized LC-MS/MS parameters used in the determination of cisplatin, carboplatin, and oxaliplatin.

Compound	Precursor Ion (m/z)	Product Ions (Quantifier/Qualifier) (m/z)	CE (eV)	Fragmentor (V)
Cisplatin	317.9	264.5/300.7	15/15	166
Carboplatin	372.0	355.0/294.0	10/20	166
Oxaliplatin	398.0	308.0/96.0	20/25	166

**Table 2:** Optimized MS/MS parameters used in the determination of platin derivatives.

Compound	Precursor Ion (m/z)	Product ions (Quantifier/Qualifier) (m/z)	CE (eV)	Fragmentor (V)	RT (min)
Pt-DDTC	492	116.0/88.0	25/25	166	5.346

**Table 3:** Quality parameters of the evaluated methods for determination of platinated drugs in urine samples.

Quality Parameter		LC-UV(DAD)	LC-MS (SIM)	Derivatization + LC-MS/MS (MRM)	LC-MS/MS (MRM)
Linearity (R^2^)	Cisplatin	0.999	0.991	0.998	0.991
	Oxaliplatin	0.999	0.996		0.992
	Carboplatin	0.998	0.991		0.994
Precision (RSD%)	Cisplatin	1.5	12	8	7.9
	Oxaliplatin	2.0	10		7.6
	Carboplatin	5.1	15		12.8
Selectivity		No (Interferences)	Yes	No (Single product as sum)	Yes
MDL (µg mL^−1^)	Cisplatin	1.5	1.5	0.0003	0.30
	Oxaliplatin	1.5	0.75		0.015
	Carboplatin	7.5	2.25		0.15
MQL (µg mL^−1^)	Cisplatin	5.0	5.0	0.001	1.0
	Oxaliplatin	5.0	2.5		0.05
	Carboplatin	25	7.5		0.50

**Table 4:** Concentration of platinum-based cytostatic drugs in urine samples from oncologic patients.

Sample	Compound	Concentration (µg mL^−1^)
01	Cisplatin	11.6
02	Cisplatin	7.34
03	Oxaliplatin	0.33
04	Cisplatin	8.43
05	Oxaliplatin	11.9
06	Carboplatin	42.5
07	Oxaliplatin	1.07
08	Carboplatin	58.7

## Data Availability

Not applicable.

## Supplementary Materials

The following supporting information can be downloaded at: https://www.mdpi.com/article/10.3390/molecules27238139/s1, Figure S1: chemical structures of cisplatin, carboplatin and oxaliplatin; Figure S2: mass spectrum of platinum-based cytostatic drugs; Figure S3: chemical structure of platinum-DDTC complex.

## Sample Availability

Samples of the compounds are available from the authors.
