# Supplementary material for: Comparison of Different Techniques for the Determination of Platinized Cytostatic Drugs in Urine Samples
Source: Molecules. 2022 Nov 23;27(23):8139. doi: 10.3390/molecules27238139 (PMC9735434; doi:10.3390/molecules27238139)
Supplement: Supplementary file 1 [file molecules-27-08139-s001.zip › molecules-1997808-supplementary.pdf]

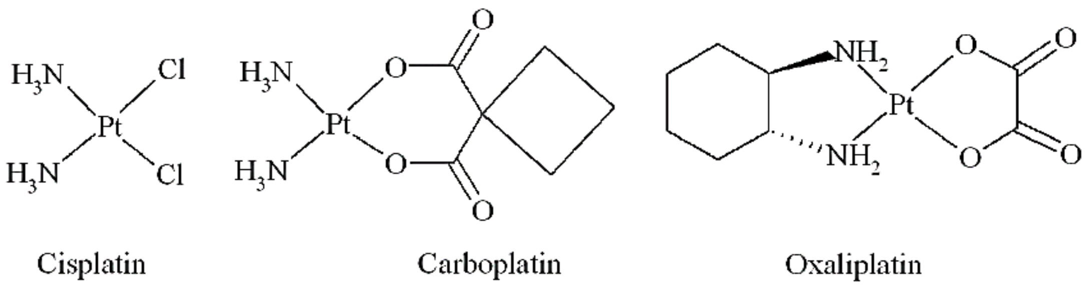


**Figure S1.** Chemical structures of cisplatin, carboplatin and oxaliplatin.

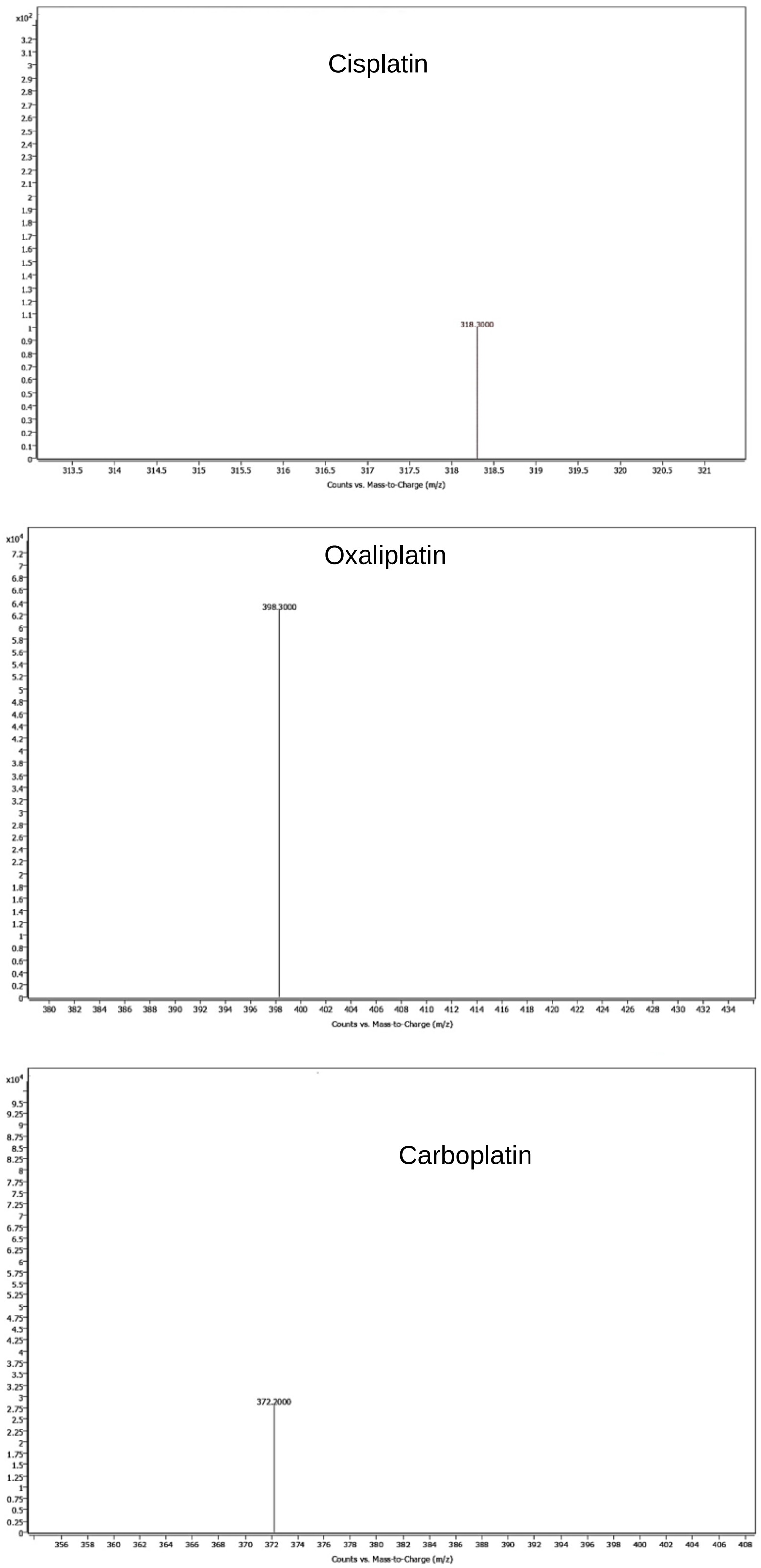


**Figure S2.** Mass spectrum of platinum-based cytostatic drugs.

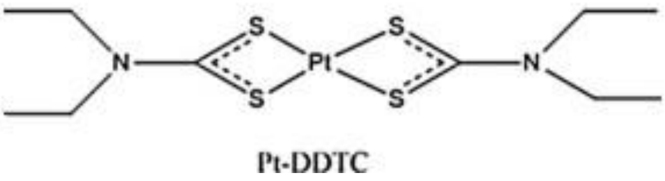


**Figure S3.** Chemical structure of platinum-DDTC complex.
